# Draft Genome Sequence of *Streptomyces* sp. Strain AM-2504, Identified by 16S rRNA Comparative Analysis as a Streptomyces kasugaensis Strain

**DOI:** 10.1128/MRA.00966-19

**Published:** 2019-09-19

**Authors:** Valerio Napolioni, Lucia Cimarelli, Antonino Miano, Anna La Teana, Retina Çapuni, Anna Maria Giuliodori, Attilio Fabbretti, Roberto Spurio

**Affiliations:** a Department of Neurology and Neurological Sciences, Stanford University School of Medicine, Stanford, California, USA; b Laboratory of Genetics, School of Biosciences and Veterinary Medicine, University of Camerino, Camerino, Italy; c Department of Life and Environmental Science, Polytechnic University of Marche, Ancona, Italy; d Structural Biology Unit, CIC bioGUNE, Derio, Bizkaia, Spain; Georgia Institute of Technology

## Abstract

We report here the draft genome sequence of *Streptomyces* sp. strain AM-2504, a microorganism producing a broad range of biotechnologically relevant molecules. The comparative analysis of its 16S rRNA sequence allowed the assignment of this strain to the Streptomyces kasugaensis species, thus fostering functional characterization of the secondary metabolites produced by this microorganism.

## ANNOUNCEMENT

*Streptomyces* is a genus of Gram-positive filamentous-growth bacteria belonging to the phylum *Actinobacteria*. *Streptomyces* bacteria are of great biotechnological interest because they produce several enzymes (e.g., cellulase, protease, pectinase, xylanase, chitinase, and amylase) used in the manufacturing industry (e.g., paper, detergents, food) and bioactive molecules widely used in both the medical and agricultural fields, such as antibiotics, anticancer drugs, immunosuppressants, antifungals, and antiparasitic drugs (1, 2).

*Streptomyces* sp. strain AM-2504 was isolated from soil, and it was originally characterized by Ōmura et al. (3) in the 1970s. This *Streptomyces* sp. strain can produce a wide range of antibiotics, such as aureothricin, thiolutin (3), dityromycin (3–6), and virantmycin B and C (7).

Spores of *Streptomyces* sp. strain AM-2504, provided by Satoshi Ōmura (Kitasato University, Tokyo, Japan), were grown in a medium containing yeast extract (0.1%), MgCl_2_ (6H_2_O) (0.1%), tryptone soya (0.3%), and sucrose (10%) at 28°C for 18 h with aeration. Genomic DNA of *Streptomyces* sp. strain AM-2504 was extracted using a chromosomal DNA extraction kit (RBC Real Genomics), while sequencing libraries were prepared using a Nextera XT kit (Illumina, CA, USA) according to the manufacturer’s instructions. Whole-genome sequencing was performed at a commercial service provider (BMR Genomics, Italy) on an Illumina MiSeq platform using a MiSeq version 3 600-cycle kit, yielding a total of 1,207,572 (2 × 300-bp-long) paired-end reads. Sequencing adapters were trimmed using Trimmomatic version 0.36.5 (8), and read quality was assessed using FastQC version 0.11.7. Prior to *de novo* assembly using Unicycler version 0.4.6.0 (9), paired reads were merged. Assembly quality was checked with QUAST version 4.6.3 (10). Genome annotation was performed with the NCBI Prokaryotic Genome Automatic Annotation Pipeline (PGAAP) software tool version 4.6 (11).

The genome assembly yielded 962 contigs with an *N*_50_ value of 15,111 bp, the largest contig having 64,571 bp. The genome of *Streptomyces* sp. strain AM-2504 consists of 8,649,584 bp with a GC content of 71.4%, a value highly similar to those reported for other *Streptomyces* species (genome sizes of 8.7 to 11.9 Mb [12] and GC content estimated at 74% [13]).

The *Streptomyces* sp. strain AM-2504 genome contains 7,788 predicted genes, including 73 RNA genes (3 complete rRNA operons, 67 tRNAs, and 3 noncoding RNAs [ncRNAs]). A total of 7,715 coding sequences (CDS), 6 clustered regularly interspaced short palindromic repeats (CRISPRs), and 405 pseudogenes were predicted using the NCBI Prokaryotic Genome Automatic Annotation Pipeline (PGAAP).

The overall genome analysis performed using autoMLST (http://automlst.ziemertlab.com), according to the average nucleotide identity (ANI) analysis, showed that *Streptomyces* sp. strain AM-2504 can be classified either as a Streptomyces celluloflavus strain (RefSeq assembly accession number GCF_000720995; ANI, 98.6%) or as a Streptomyces kasugaensis strain (RefSeq assembly accession number GCF_002261115; ANI, 98.6%). However, BLAST analysis of the 16S rRNA gene sequence identified 100% similarity with Streptomyces kasugaensis strain M338-M1 (GenBank accession number NR_024724). Bioinformatics analyses using antiSMASH version 5.0.0 (14) predicted a repertoire of 45 biosynthetic gene clusters for secondary metabolite production. Default parameters were used for all software unless otherwise noted.

Overall, these results can stimulate a wide range of approaches, from biochemistry to genetics and biotechnology, aimed at the isolation and identification of new biologically active compounds, including antibiotic agents, produced by *Streptomyces* sp. strain AM-2504.

### Data availability.

The complete genome sequence described here has been deposited in NCBI GenBank under BioProject number PRJNA523451, BioSample number SAMN10977544, GenBank accession number SIXH00000000 (version number SIXH01000000), and SRA number SRP217889.
